# Association of mentally-passive and mentally-active sedentary behaviors with device-measured bouts and breaks of sedentary time in adolescents

**DOI:** 10.34172/hpp.2021.14

**Published:** 2021-02-07

**Authors:** André O Werneck, Marcelo Romanzini, Danilo R. Silva, Adewale L. Oyeyemi, Maria R. O. Bueno, Enio R. V. Ronque

**Affiliations:** ^1^Department of Nutrition, School of Public Health, Universidade de São Paulo (USP), São Paulo; Brazil; ^2^Physical Activity and Health Laboratory, Department of Physical Education, Universidade Estadual de Londrina, Londrina, Brazil; ^3^Departament of Physical Education, Universidade Federal de Sergipe - UFS, São Cristóvão, Brazil; ^4^Department of Physiotherapy, College of Medical Sciences, University of Maiduguri, Borno State, Nigeria

**Keywords:** Exercise, Sedentary lifestyle, Accelerometry, Adolescent, Sitting position

## Abstract

**Background:** Our aim was to analyze the association of self-reported mentally-passive and mentally-active sedentary behaviors with different patterns (bouts and breaks) of device-measured sedentary time in adolescents.

**Methods:** This was a cross-sectional study conducted among 375 adolescents (177 boys) aged 1015 years. Total time, bouts and breaks of sedentary time were measured through accelerometers. Self-reported sedentary behavior in different activities was summed and divided into mentally-active (playing electronic games, studying and reading) and mentally-passive (watching TV, watching DVD, and using computer for leisure). Bayesian linear regression models were used for association analyses.

**Results:** Only mentally-passive sedentary behaviors were positively associated with longer bouts [1-4 minutes: mean posterior distribution: -0.431 (95% credible interval: -0.745 to -0.114); =15 minutes: 0.641 (0.122 to 1.222)] and lower number of breaks [-0.138 (-0.228 to -0.044)] of device-measured sedentary time.

**Conclusion:** Self-reported mentally-passive sedentary behaviors are associated with longer bouts and lower breaks of device-measured sedentary time.

## Introduction

Sedentary behavior, characterized as any waking behavior while in sitting and reclining position with an energy expenditure of ≤1.5 metabolic equivalents of task,^1^ has been consistently associated with several negative health outcomes such as obesity, metabolic and mental disorders across the life span.^2-4^ However, not only the total sedentary time, but the pattern and use of this time have been specifically associated with health and cognitive outcomes.^5^

Recent studies found that when sedentary behavior is divided into two categories according to expected cognitive demand (mentally-active and mentally-passive), only mentally-passive sedentary behavior was associated with poor mental health, while mentally-active was not associated or could even be a protective factor.^6,7^ This specific relationship may be explained by the cognition pathway, considering that cognitive activities are decreased by the length of exposure to mentally-passive sedentary behavior.^8^ In this sense, mentally-passive sedentary behavior activities as TV-viewing are associated with lower cognition, while mentally-active activities can be associated with higher cognition levels.^8,9^ The relationship could also be explained by occupational satisfaction, considering that the highest amount of mentally-active sedentary behavior is spent during and/or related with occupation.^10^

Given the potential association between sedentary behavior patterns (length of bouts or number of breaks) and cardiovascular risk factors such as metabolic risk and obesity,^4,11,12^ it could be hypothesized that the specific use of sedentary time may be a potential pathway to understanding the link between sedentary behavior and health outcomes. Here, we propose that different types of sedentary behavior (mentally-passive and mentally-active) can be accumulated in different patterns. Sedentary behaviors that require minimal cognitive efforts (mentally-passive) can be accumulated in higher bouts, while mentally-active behavior can be accumulated with fewer or higher number of breaks. However, these assumptions have not tested in previous studies among adolescents. Previous studies among older adults found that passive sedentary behaviors as TV-viewing and computer use during leisure-time were associated with prolonged bouts of sedentary time.^13^ This type of investigation is particularly important among adolescents as it could inform specific strategies and interventions on the type of activity and pattern of behavior. Therefore, our aim was to analyze the association of self-reported mentally-active and mentally-passive sedentary behavior with different patterns of device-measured sedentary time (bouts and breaks) among adolescents.

## Materials and Methods

### Participants

This was a school-based cross-sectional study, conducted between October 2015 and May 2017, in primary public schools in Londrina, Paraná, Brazil, involving adolescents of both sexes, aged between 10 and 14 years old. Regarding the sample process, all the public schools of the city were first divided into regions (north, south, east, west and center), and two schools were randomly selected from each region. Classes were then randomly selected from schools and all the students in these classes were invited to participate in the study. The inclusion criteria were 1) Be regularly enrolled in the 6^th^ year of elementary school, 2) Willing to wear accelerometer for at least seven consecutive days and 3) Delivery of the signed informed consent form by the legal guardian. Adolescents were excluded from the sample if they reported using prescribed medicines or were being treated for a disease. The sample size was estimated considering eight participants per correlate plus 50 participants.^14^ As the initial aim of the project was to estimate correlates of sedentary time, 47 correlates were included, resulting in a minimal sample of 426 adolescents. However, only 375 adolescents presented valid accelerometer data. From 680 initial participants, 286 did not present valid accelerometer data (failed to provide minimal cutoff points for valid data) and five adolescents presented missing data on the covariates and, therefore, were excluded from final sample.

### Self-reported and device-based sedentary behavior

ActiGraph (ActiGraph, Pensacola, FL, USA) GT3X and GT3X-Plus models were used to assess device-measured sedentary time patterns. Participants were asked to wear the accelerometer on the right side of the hip for seven consecutive days; only to be removed during shower, aquatic activities and sleep time. For the present study, 15 s epochs were used (ActiLife software, version 6.8.2). Adolescents with at least four valid days (>480 min/d, with at least one weekend day) registered by the accelerometer were included in the analyses.^15^ The criterion of 60 minutes of consecutive zeros was utilized to determine the non-wear time.^15^ Sedentary behavior was classified using cut-points developed for ActiGraph vector magnitude counts (180 counts.15s-1) in Brazilian adolescents.^16^ Bouts are defined as uninterrupted periods in sedentary behavior^17^ (drop time = 0) with durations of 1-4 minutes, 5-14 minutes and ≥15 minutes. Breaks were defined as the non-sedentary period between two sedentary bouts.^17^ For analytic purposes, total sedentary time and time accumulated in bouts were expressed as percentage values (% of total time using accelerometer), while breaks were expressed as mean frequency by hour (breaks.hour-1).

Domains of self-reported sedentary behavior were assessed through general questions about different sedentary behaviors: “Considering a typical weekday (Monday to Friday), how much time do you spend… (e.g. watch TV)” and “Considering a typical weekend day (Saturday and Sunday), how much time do you spend… (e.g. watch TV)”. These questions were asked for watching tv, watching DVD, using computer for leisure, playing electronic games, studying and reading, with 6 possible answers: (a) none, (b) less than 1 hour, (c) between 1 and 2 hours, (d) between 2.01 and 3 hours, (e) between 3.01 and 4 hours, (f) more than 4 hours. Mean time spent in each behavior was computed (e.g. less than 1 hour was transformed to 0.5 hours) and behaviors were divided into mentally-passive (watching TV, watching DVD, using computer for leisure) and mentally-active (playing electronic games, studying and reading) according to the expected cognitive demand. The mean time in each categories of sedentary behavior was summed,^6^ and two continuous indicators were created. The reproducibility was tested using a sample of 25 adolescents with similar characteristics, which were not included in the final sample. We found the following reproducibility, with a one-week interval, for the questions (in intra-class correlation coefficients - ICC): watching TV = ICC: 0.90, watching DVD = ICC: 0.33, using computer for leisure = ICC: 0.72, playing electronic games = ICC: 0.54, studying = ICC: 0.87 and reading = ICC: 0.79.

### Covariates

Sex, chronological age, somatic maturation (estimated through the estimated age at peak height velocity [PHV]^18^), cardiorespiratory fitness (Léger 20-m shuttle run test), and body mass index (through measures of stature and height), were adopted as covariates. Socioeconomic status was assessed through the ABEP questionnaire.^19^

### Statistical analysis

Descriptive statistics were presented with mean and 95% confidence intervals. For the association analysis, we created Bayesian linear regression models, having in mind the limitations of frequentist approach, to explore the associations of self-reported mentally-passive and mentally-active sedentary behaviors (main exposures) with different bouts (1-4 minutes, 5-14 minutes and ≥15 minutes) and breaks of device-measured sedentary time (treated as outcomes). Mean posterior distribution and the respective 95% credible intervals were used. All analyses were performed using the software Stata 15.1 (StataCorp. 2017. Stata Statistical Software: Release 15.1. College Station, TX: StataCorp LLC).

## Results

Our final sample was composed of 375 adolescents (177 boys), aged between 10.3 and 14.5 years. Characteristics of sample are presented in Table 1. In general, self-reported sedentary behaviors were similar between boys and girls. Adolescents reported around 4.2 h/d of mentally-passive sedentary behavior and 3.2 h/d of mentally-active sedentary behavior.

The association between different types of self-reported sedentary behavior and bouts and breaks of sedentary time is shown in Table 2. Mentally-passive activities were positively associated with longer bouts of sedentary time. The main associations were found for DVD-viewing [1-4 minutes: mean posterior distribution: -0.925 (95% credible interval: -1.674 to -0.182); ≥15 minutes: mean posterior distribution: 1.542 (95% credible interval: 0.306 to -2.754)] and computer use for leisure [1-4 minutes: mean posterior distribution: -0.974 (95% credible interval: -1.554 to -0.324); ≥15 minutes: mean posterior distribution: 1.562 (95% credible interval: 0.503 to 2.586)]. Similarly, the overall score of mentally-passive sedentary behavior was associated with longer bouts [1-4 minutes: mean posterior distribution: -0.431 (95% credible interval: -0.745 to -0.114); ≥15 minutes: mean posterior distribution: 0.641 (95% credible interval: 0.122 to 1.222)] and lower number of breaks [mean posterior distribution: -0.138 (95% credible interval: -0.228 to -0.044) of device-measured sedentary time. On the other hand, mentally active sedentary behaviors were not consistently associated with patterns of device-measured sedentary time.

## Discussion

Our aim was to analyze the association of mentally-passive and mentally-active self-reported sedentary behavior with device-measured sedentary time patterns. Our main findings were that self-reported mentally-passive sedentary behavior was associated with longer bouts and lower number of breaks in sedentary time. Self-reported mentally-active sedentary behavior was consistently not associated with patterns of device-measured sedentary time. To our knowledge, this was the first study to investigate the associations of types of self-reported sedentary behavior and patterns of device-measured sedentary time among adolescents.

Sedentary behavior, among adolescents, is associated with several negative health outcomes such as obesity,^4^ metabolic syndrome risk,^2^ lower cognition^8^ and depressive symptoms.^3^ However, previous findings also reported that different types of sedentary behaviors can have different roles in the association with mental health,^5^ given that mentally-passive behaviors such as TV-viewing have been especially associated with poorer indicators of mental health. Recently, studies found that mentally-passive sedentary behavior was associated with higher rates of depression among adults, while mentally-active sedentary behavior was not consistently associated or even protective of depression.^6,7^ The cognitive demands and occupational pathways have been used to explain these findings between different type of sedentary behavior and mental health outcomes.^10,20^

Beyond the total time and type of activity, the pattern of sedentary behavior is associated with health outcomes.^4,21^ Thus, our finding could be an alternative potential mechanism that can be used to explain and understand the nexus between sedentary behaviors and health outcome. The present finding suggests that breaks and bouts of sedentary time might be potential variables that can be explored in future studies as the mediators of the association between self-reported mentally-passive sedentary behaviors and health outcomes.

Higher uninterrupted time in sedentary behavior is associated with several alterations on metabolism, including the elevation of inflammatory levels, especially due to lack of muscle contraction and consequently release of anti-inflammatory hormones.^22^ In this pathway, elevated inflammatory cytokines levels are associated with poorer mental health indicators.^23^ Also, longer bouts are associated with greater levels of adiposity,^4^ which is also associated with poorer mental health.^24^ Current findings should be considered for further studies on the association between sedentary behavior and health outcomes. Possibly, the negative impact of sedentary behavior, especially on mental health, could be better explained when the patterns and cognitive demand of sedentary behaviors or even their interactions are taken into consideration in future studies.

Our findings highlight that future interventions should be focused on reducing mentally-passive sedentary behavior activities, such as TV-viewing, computer using for leisure and DVD viewing. To achieve the reduction of sedentary behavior, interventions should explore the potential determinants of the specific behaviors aiming to create different strategies and priority groups to focus on different types and manifestations of sedentary behavior.^25^ For example, computer use is higher among older adolescents, while TV-viewing is similar among younger and older adolescents.^26^ Also, there are several social (e.g. parental sedentary behavior) and environmental (e.g. availability of electronic devices as television inside the bedroom) determinants associated with longer mentally-passive sedentary behavior.^27,28^

Our results should be interpreted in the light of the potential limitations. First, due to a limitation from the questionnaire, the maximum amount of time answer for each type of sedentary behavior was 4 h/d, which can be a potential bias. However, the prevalence of the highest category was low. Second, we were not able to adjust the analyses for other potential mediators/confounders as inflammation and cognition. However, this is the first study to explore this new pathway between device-measured and self-reported measures of sedentary behavior.

## Conclusion

Thus, self-reported mentally-passive sedentary behavior was associated with longer periods of uninterrupted sedentary time, while self-reported mentally-active sedentary behavior was not associated with device-measured sedentary time among adolescents. The relationship between pattern and cognitive demand of sedentary time should be confirmed in other population groups in order to guide the interpretation of the studies linking sedentary behavior and health outcomes.

## Acknowledgements

We gratefully acknowledge the contributions of all participants of the present research as well as the staff for the data collection. André O. Werneck is supported by the São Paulo Research Foundation (FAPESP) with a PhD scholarship (FAPESP process: 2019/24124-7). Enio RV Ronque is funded by National Council for Scientific and Technological Development - Brazil (CNPq) with a research productivity grant (CNPq process: 309731/2018-6).

## Funding

This research received no specific grant from any funding agency in the public, commercial, or not-for-profit sectors.

## Competing interests

The authors report no conflicts of interest.

## Ethics approval

All procedures performed in the original studies involving human participants were approved by Londrina State University Ethics Committee (process: 1.281.324) in accordance with the ethical standards of the institutional and/or national research committee and with the 1964 Helsinki Declaration and its later amendments or comparable ethical standards. Additionally, informed consent was obtained from all individual participants as well as the parents of included participants.

## Authors’ contributions

AOW: Concept and study design, data analysis, interpretation of the data and drafted the initial manuscript. MR, MROB and ERVR: Conceptualization, study design, collection of data, supervision and critical revision. DRS and ALO: Critical revision and approval of the manuscript with important intellectual content. All authors have read and approved the final version of the manuscript and agree with the order of presentation of the authors.

**Table 1:** Characteristics of sample according to sex

	**Boys** **(n = 177)**	**Girls** **(n = 198)**
Chronological age, y	11.9 (11.8 to 12.0)	11.8 (11.7 to 11.9)
Body mass index, kg/m^2^	20.0 (19.3 to 20.6)	19.9 (19.3 to 20.5)
Age at PHV, y	13.6 (13.6 to 13.7)	11.9 (11.8 to 12.0)
Socioeconomic status, score	4.6 (4.4 to 4.7)	4.4 (4.2 to 4.5)
Sedentary time, %	69.6 (68.4 to 70.8)	70.1 (69.1 to 71.2)
Bouts, % of sedentary time		
1-4 minutes	29.4 (28.2 to 30.6)	30.9 (29.8 to 32.0)
5-14 minutes	29.1 (28.2 to 30.0)	28.1 (27.4 to 28.8)
≥ 15 minutes	30.9 (28.8 to 32.9)	29.3 (27.5 to 31.0)
Breaks in sedentary time, n/h	11.2 (10.9 to 11.6)	11.7 (11.4 to 12.0)
TV-viewing, h/d	1.7 (1.5 to 1.9)	1.9 (1.8 to 2.1)
DVD-viewing, h/d	1.2 (1.0 to 1.4)	1.1 (0.9 to 1.2)
Computer use for leisure, h/d	1.5 (1.3 to 1.7)	0.9 (0.7 to 1.0)
Playing electronic games, h/d	1.9 (1.7 to 2.1)	1.4 (1.3 to 1.6)
Studying, h/day	0.5 (0.4 to 0.7)	0.6 (0.5 to 0.7)
Reading, h/day	0.5 (0.4 to 0.7)	0.6 (0.5 to 0.7)
Mentally-passive SB, h/d	4.4 (4.0 to 4.9)	3.9 (3.5 to 4.2)
Mentally-active SB, h/d	3.4 (3.0 to 3.8)	3.0 (2.7 to 3.3)

Abbreviations:PHV, peak of height velocity. SB, sedentary behavior. *Note*. Values are presented using values of mean and 95% confidence interval.

**Table 2:** Bayesian linear regression models of the association of self-reported passive and mentally-active sedentary behaviors with device-measured sedentary time

	**Sedentary time, %**	**Bouts of sedentary behavior**			**Breaks in sedentary time, n/h**
		**1-4 minutes**	**5-14 minutes**	**≥ 15 minutes**	
TV-viewing, h/d	0.373 (-0.277 to 1.043)	-0.192 (-0.796 to 0.420)	0.389 (-0.037 to 0.808)	0.080 (-0.939 to 1.153)	-0.086 (-0.272 to 0.096)
DVD-viewing, h/d	0.502 (-0.247 to 1.253)	-0.925 (-1.674 to -0.182)	-0.375 (-0.848 to 0.143)	1.542 (0.306 to 2.754)	-0.286 (-0.511 to -0.051)
Computer use for leisure, h/d	1.220 (0.580 to 1.839)	-0.974 (-1.554 to -0.324)	0.008 (-0.450 to 0.518)	1.562 (0.503 to 2.586)	-0.317 (-0.503 to -0.130)
Playing electronic games, h/d	0.779 (0.290 to 1.286)	-0.517 (-1.093 to 0.050)	0.307 (-0.078 to 0.672)	0.637 (-0.233 to 1.561)	-0.161 (-0.336 to 0.005)
Studying, h/d	-0.140 (-0.998 to 0.729)	0.206 (-0.686 to 1.097)	-0.406 (-1.010 to 0.189)	0.101 (-1.412 to 1.561)	0.025 (-0.247 to 0.294)
Reading, h/d	-0.125 (-1.011 to 0.775)	0.188 (-0.698 to 1.112)	-0.417 (-1.081 to 0.234)	0.063 (-1.319 to 1.567)	0.025 (-0.236 to 0.299)
Mentally-passive SB, h/d	0.447 (0.169 to 0.733)	-0.431 (-0.745 to -0.114)	0.014 (-0.185 to 0.235)	0.641 (0.122 to 1.222)	-0.138 (-0.228 to -0.044)
Mentally-active SB, h/d	0.195 (-0.108 to 0.495)	-0.014 (-0.368 to 0.311)	-0.026 (-0.278 to 0.195)	0.087 (-0.469 to 0.650)	-0.019 (-0.117 to 0.076)

Note. Values are presented in mean predicted posterior distribution and 95% credible intervals. Adjusted for sex, chronological age, age at peak height velocity and body mass index.
